# Prevalence of sleep disorders among first responders for medical emergencies: A meta-analysis

**DOI:** 10.7189/jogh.12.04092

**Published:** 2022-10-20

**Authors:** Garry Huang, Tso-Ying Lee, Kondwani Joseph Banda, Li-Chung Pien, Hsiu-Ju Jen, Ruey Chen, Doresses Liu, Shu-Tai Shen Hsiao, Kuei-Ru Chou

**Affiliations:** 1 School of Health Care Administration, College of Management, Taipei Medical University, Taipei, Taiwan; 2 Australasian College of Paramedicine, Australia; 3 Australian Institute of Project Management, Australia; 4 Nursing Research Center, Department of Nursing, Taipei Medical University Hospital, Taipei, Taiwan; 5 School of Nursing, College of Nursing, Taipei Medical University, Taipei, Taiwan; 6 Endoscopy Unit, Surgery Department, Kamuzu Central Hospital, Lilongwe, Malawi; 7 Post-Baccalaureate Program in Nursing, College of Nursing, Taipei Medical University, Taipei, Taiwan; 8 Psychiatric Research Center, Wan Fang Hospital, Taipei Medical University, Taipei, Taiwan; 9 Department of Nursing, Taipei Medical University-Shuang Ho Hospital, New Taipei, Taiwan; 10 Department of Nursing, Wan Fang Hospital, Taipei Medical University; 11 Center for Nursing and Healthcare Research in Clinical Practice Application, Wan Fang Hospital, Taipei Medical University, Taipei, Taiwan; 12 Superintendent Office, Taipei Medical University Hospital, Taipei, Taiwan; 13 Psychiatric Research Center, Taipei Medical University Hospital, Taipei, Taiwan; 14 Neuroscience Research Center, Taipei Medical University, Taipei, Taiwan

## Abstract

**Background:**

Shift work and irregular work schedules among first responders have been associated with physical and psychological problems such as sleep disorders. We conducted the first meta-analysis to explore and estimate the prevalence of sleep disorders among first responders for medical emergencies.

**Methods:**

We searched four databases: Web of Science, Psych Info, CINAHL, and PubMed. The Generalized Linear Mixed model (GLMM) was used to estimate the prevalence estimates of sleep disorders in R software and the DerSimonian-Lard random-effects model in Comprehensive Meta-Analysis was used to explore associated comorbidities for OSA and insomnia, presented as odds ratios (ORs) and confidence intervals (CIs). The Cochran's Q, τ^2^, and the statistics were used to assess heterogeneity and the moderator analysis was conducted to identify moderator variables.

**Results:**

Twenty-eight studies with 100 080 first responders were included from the total of 1119 studies retrieved from the databases. The prevalence rates for sleep disorders were 31% (95% CI = 15%-53%) for shift work disorder (SWD), 30% (95% CI = 18%-46%) for obstructive sleep apnea (OSA), 28% (95% CI = 19%-39%) for insomnia, 28% (95% CI = 24%-33%) for excessive daytime sleepiness (EDS), 2% (95% CI = 1%-4%) for restless leg syndrome, and 1% (95% CI = 0%-5%) for narcolepsy. Anxiety (OR = 2.46; 95% CI = 1.99%-3.03%), cardiovascular disease (CVD) (OR = 2.03; 95% CI = 1.43-2.88), diabetes mellitus (DM) (OR = 1.93; 95% CI = 1.41-2.65), depression (OR = 1.89; 95% CI = 1.01-3.56), gastroesophageal reflux disease (GERD) (OR = 1.83; 95% CI = 150-2.22), and post-traumatic stress disorder (PTSD) (OR = 1.78; 95% CI = 1.33-2.39) were associated with OSA. Depression (OR = 9.74; 95% CI = 4.67-20.3), anxiety (OR = 9.22; 95% CI = 3.81-22.3), and PTSD (OR = 7.13; 95% CI = 6.27-8.10) were associated with insomnia. Age, gender, first responders, continent, study quality, study design, and assessment tool were significant moderator variables for OSA, insomnia, and EDS.

**Conclusions:**

This meta-analysis found a substantially high prevalence of sleep disorders including SWD, OSA, insomnia, and EDS among first responders for medical emergencies. Early assessment and management of sleep disorders among first responders is necessary to promote good, quality sleep to help prevent anxiety, depression, CVD, DM, GERD, and PTSD.

Sleep is a biological function necessary for vital functions, including memory, emotion, learning, metabolic regulation, cellular toxin removal, and neural development in humans [1]. It is crucial in maintaining survival, recovery, and energy conservation, which are important for good health and improved quality of life, while poor sleep has severe health implications as it interrupts circadian rhythms [1,2]. Circadian rhythms are responsible for regulating the body’s processes (i.e., the behavioural, physical, and mental changes that occur over 24 hours) through the release of melatonin [3]. Poor sleep habits have been associated with short-term effects including irritability, difficulty with concentration, headaches, and mood changes and long-term effects including increased risk for cardiovascular disease, cancer, and obesity [4,5]. Thus, exploring sleep problems among first responders is crucial for preventing physical and mental problems leading to improved service delivery among this population.

First responders are a group of professionals that includes emergency medical service (EMS) personnel including ambulance personnel, paramedics, and emergency medical technicians (EMTs), police, and fire fighters [6-8]. These frontline workers are responsible for pre-hospital medical services during emergencies, accidents, and disasters facing numerous calls and irregular daily schedules that disturb their usual sleep patterns leading to sleep disorders, which have been associated with physical and mental health disorders [4,9]. Sleep disorders lead to disturbance of normal behaviour or usual sleeping patterns [10]. The International Classification of Sleep Disorders, Third Edition (ICSD-3) and International Classification of Diseases, Tenth Edition (ICD-10) define, classify, and categorize sleep disorders into those of organic origin focusing on medical and neurological-based disorders and non-organic origin focusing on behavioural and mental disorders. The ICSD-3 is the most advanced classification of sleep disorders that categorizes sleep disorders into 10 conditions including restless legs syndrome, insomnia, nightmare disorder, hypersomnolence, breathing-related sleep disorders, narcolepsy, non-rapid eye movement sleep (NREM) sleep arousal disorders, substance- or medication-induced sleep disorders, circadian rhythm sleep awake disorders, and rapid eye movements (REM) sleep behaviour disorder.

Current evidence on the prevalence of sleep disorders among first responders for medical emergencies remains unclear and unknown in the current research. A systematic review by Jones [11] showed that sleep disturbances were associated with poor alertness and concentration, fatigue, mood disturbances, cognitive impairment, and poor quality of life among first responders. Moreover, demographic factors (education, gender, age, smoking status, alcohol status, living habits and environment, marital status, mental disorders, and medical history) and occupational factors (irregular work schedules, type of work, profession, peer support, and awareness of support measures) have shown to have an impact on the occurrence of sleep disorders [4]. However, previous systematic review included limited number of studies, explored no specific sleep disorders but focused on sleep disturbances, and found no conclusive evidence on the prevalence of sleep disorders. Thus, no comprehensive meta-analysis has been conducted to explore and estimate the prevalence of sleep disorders among first responders for medical emergencies, revealing a knowledge gap that needs to be addressed. To address this and extend current evidence by conducting a first meta-analysis, we aimed to investigate and estimate the prevalence of sleep disorders and associated comorbidities among first responders for medical emergencies. We hypothesized that first responders for medical emergencies would demonstrate similar prevalence rates of sleep disorders to that of other shift workers.

## METHODS

### Search strategy

The study protocol was registered with PROSPERO: CRD42022330754 and followed the Meta-Analysis of Observational Studies in Epidemiology (MOOSE) guidelines [12]. We comprehensively searched PubMed, Web of Science, CINAHL, and Psych Info from each database date of inception with initial search on 20/02/ February 20, 2022, and a follow-up search on May 11, 2022. The following keywords were used in combination with detailed search strategy including MeSH terms in Table S1 in the **Online Supplementary Document**: (prevalence OR incidence OR epidemiology OR rate OR rates OR number OR proportion OR probability OR event) AND (insomnia OR narcolepsy OR REM sleep behavior disorder OR restless leg syndrome OR parasomnias OR sleep apnea OR Non-24-hour sleep wake disorder OR excessive sleepiness OR shift wake disorder OR periodic limb movement disorder) AND (Emergency Medical Services Personnel OR EMS personnel OR ambulance personnel OR fire fighters OR law enforcement OR police OR first responders OR paramedics OR emergency medical technicians OR EMTs). A manual search was conducted by reviewing the reference lists of relevant observational studies, systematic review, and meta-analysis and potential studies were then searched in Google. We also contacted original study authors for missing data to ensure most eligible studies were included.

### Study selection

**Figure 1:**
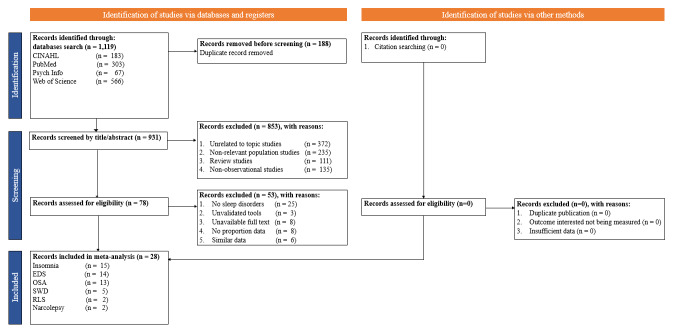
Flowchart for study selection.

The eligible studies inclusion was based on the PICOS criteria without any language restrictions were: 1) population: first responders including emergency medical service (EMS) personnel, ambulance personnel, paramedics, police, emergency medical technicians (EMTs), and fire fighters; 2) exposure of interest: sleep disorders (insomnia, narcolepsy, REM sleep behaviour disorder, restless leg syndrome, parasomnias, sleep apnoea, non-24-hour sleep wake disorder, excessive sleepiness, shift wake disorder, periodic limb movement disorder); 3) comparison: no sleep disorders, 4) outcome of interest: epidemiology, incidence, or prevalence using a validated assessment tool, and 5) study design: observational studies including cross-sectional and prospective studies (Table S2 in the **Online Supplementary Document**). Studies were excluded if they were non-relevant population studies, systematic review or meta-analysis studies, studies using non-validated assessment tools, study protocols, duplicate studies, studies unrelated to the topic, and randomized controlled trials (**Figure 1**).

### Data extraction and study outcomes

Two independent reviewers (GH and KJB) extracted the data into following categories: 1) author and year of publication, 3) age, 4) sample size, 5) gender, 6) study design, 7) country, 8) continent, 9) sleep disorders including obstructive sleep apnoea (OSA), restless leg syndrome (RLS), shift work disorder (SWD), insomnia, excessive daytime sleepiness (EDS), and narcolepsy, 10) assessment tool, and 11) associated comorbidities, including anxiety, depression, cardiovascular disease (CVD), diabetes mellitus (DM), gastroesophageal reflux disease (GERD), and post-traumatic stress disorder (PTSD) in the included studies.

The primary outcome was pooled prevalence of sleep disorders, including 1) SWD measured by Shift Work Disorder Screening Questionnaire (SWDSQ) and International Classification of Sleep Disorders-2nd Edition (ICSD-2), 2) OSA measured by polysomnography using Apnea-Hypopnea Index (AHI), Berlin Questionnaire (BQ), and snoring, tiredness, observed apnoea, blood pressure, body mass index, age, neck size, gender (STOP-BANG) questionnaire, 3) insomnia measured by Athens Insomnia Scale (AIS) and Insomnia Severity Index (ISI), 4) EDS measured by Epworth Sleepiness Scale (ESS), 5) RLS measured by Restless Leg Syndrome Epidemiology, Symptoms and Treatment Questionnaire (RLSESTQ), and 6) narcolepsy among first responders. The secondary outcomes were associated comorbidities including anxiety, depression, CVD, DM, GERD, and PTSD for OSA and insomnia (**Table 1**).

**Table 1:** Demographic characteristics for first responders with sleep disorders

				Gender (n)				
**Author (year)**	**Country**	**Mean age (years)**	**Sample size (N)**	**Male**	**Female**	**Other**	**Study design**	**Study quality**
Angehrn et al. (2020) [13]	Canada	39.0	4019	NA	NA	4019	Cross-sectional	8 – M
Barger et al. (2015) [14]	USA	40.4 (±8.9)	6933	6360	410	163	Cross-sectional	9 – L
Cramm et al. (2021) [15]	Canada	40.0	1217	1024	77	116	Cross-sectional	8 – M
Fernandez et al. (2014) [16]	USA	36.9	1047	703	255	89	Cross-sectional	10 – L
Garbarino et al. (2002) [17]	Italy	29.7 (±5.6)	1280	1060	220	NA	Cross-sectional	7 – M
Garbarino et al. (2019) [18]	Italy	36.0 (±7.4)	236	236	NA	NA	Cross-sectional	7 – M
Glaser et al. (2014) [19]	USA	51.5 (±7.9)	636	636	NA	NA	Cross-sectional	8 – M
Haddock et al. (2013) [20]	USA	38.2 (±9.9)	458	458	NA	NA	Cross-sectional	8 – M
Hendrickson et al. (2022) [21]	USA	39.6 (±1.1)	187	NA	NA	187	Cross-sectional	7 – M
Jang et al. (2020) [22]	South Korea	40.0	9,738	9080	658	NA	Cross-sectional	9 – L
Khan et al. (2020) [23]	Australia	39.1 (±12.1)	136	62	74	NA	Cross-sectional	7 – M
Khan et al. (2020) [24]	Australia	44.1 (±12.1)	83	83	NA	NA	Cross-sectional	7 – M
Khan et al. (2020) [24]	Saudi Arabia	39.1 (±12.1)	104	104	NA	NA	Cross-sectional	7 – M
Kim et al. (2021) [25]	South Korea	40.7 (±9.6)	51 149	46 544	4605	NA	Cross-sectional	9 – L
Klawe et al. (2005) [26]	Poland	47.1 (±3.2)	21	NA	NA	21	Cross-sectional	7 – M
Kwak et al. (2020) [27]	South Korea	40.1 (±8.7)	352	328	24	NA	Cross-sectional	8 – M
Lim et al. (2020) [28]	South Korea	39.6 (±8.7)	9788	9124	664	NA	Cross-sectional	9 – L
Pan et al., (2019) [29]	China	40.1 (±9.1)	1036	799	237	NA	Cross-sectional	9 – L
Pazmino Erazo et al. (2020) [30]	Ecuador	30.0	27	16	11	NA	Cross-sectional	7 – M
Pinto et al. (2018) [31]	Brazil	34.6 (±6.1)	22	22	NA	NA	Cross-sectional	7 – M
Pirrallo et al. (2012) [32]	USA	35.7	1306	905	401	NA	Cross-sectional	7 – M
Rajaratnam et al. (2011) [33]	USA and Canada	38.5 (±8.3)	4957	4079	861	17	Prospective cohort	9 – L
Savall et al. (2021) [34]	France	39.1 (±11.5)	193	159	34	NA	Cross-sectional	7 – M
Shi et al. (2021) [35]	USA	42.9	268	156	112	NA	Cross-sectional	7 – M
Sofianopoulos et al. (2020) [36]	Australia	39.7	60	46	14	NA	Cross-sectional	7 – M
Sunderram et al. (2018) [37]	USA	52.8 (±9.9)	601	498	103	NA	Cross-sectional	9 – L
Tsehay et al. (2021) [38]	Ethiopia	34 (±7.4)	385	324	61	NA	Cross-sectional	8 – M
Webber et al. (2011) [39]	USA	42.3 (±8.0)	4756	NA	NA	4756	Prospective cohort	7 – M
Yadav et al. (2015) [40]	India	26.6 (±0.6)	85	34	51	NA	Cross-sectional	7 – M

F – female, h – poor quality, L – high quality, M – moderate quality, Ma – male, NA – not available, USA – United States of America

### Quality assessment of included studies

The quality of the included studies was examined by GH and KJB using the Hoy’s Risk of Bias assessment tool [41]. The assessment tool has 10 domains that assesses the internal and external validity of prevalence observational studies. Selection and non-response bias represent the external validity of the study domains assessed in items 1-4. Measurement bias and bias related to the analysis represent the internal validity of the study domains assessed in items 5-10. The 10 items with the score for each item being 1 for low risk and 0 for high risk are added to have the overall risk of study bias. The quality of the included studies was as follows: 9-10 for a low risk of bias study, 7-8 for a moderate risk of bias study, and 0-6 for a high of bias risk study (**Table 1**, Table S3 in the **Online Supplementary Document**). A third expert reviewer (KRC) resolved and addressed differences between GH and KJB through discussions.

### Statistical methods

The generalized linear mixed model (GLMM) in R-software was used to estimate the pooled estimates of sleep disorders (SWD, OSA, insomnia, EDS, RLS, and narcolepsy) among first responders [42,43]. This method combines proportions and accounts for within-study variations, accounting for studies with small sample sizes and rare events. The GLMM does not need corrections in the analysis for zero counts and models prevalence estimates with binomial likelihoods without any data transformations for each study and uses specific link function to transform proportion estimates to a linear scale. Statistical significance for all the outcomes was set at a *P*-value of <0.05.

Heterogeneity assessment of OSA, insomnia, and EDS prevalence estimates was assessed to account for variations regarding participants and methodological factors among the included studies. An *X^2^* test using Cochran's Q, τ^2^, and *I^2^* statistics (*P*<0.10) [42,44] indicating low (<25%), moderate (≥25% to ≤75%), and high (≥75%) heterogeneity was used for heterogeneity assessment. Publication bias for OSA, insomnia, and EDS was measured by the Egger’s regression test and funnel plot of the proportion estimates [45].

The DerSimonian-Lard random and fixed-effects models in Comprehensive Meta-Analysis (CMA), Version 2.0 software was used to assess the associated comorbidities for OSA and insomnia, including anxiety, depression, CVD, DM, GERD, and PTSD [46]. The inverse variance-weighted mean of the logarithm of odds ratios (ORs) with 95% confidence intervals (CIs) was used in CMA to estimate the pooled OR for associated comorbidities (anxiety, depression, CVD, DM, GERD, and PTSD) for OSA and insomnia.

Pre-specified demographic and methodological factors for OSA, insomnia, and EDS were used for moderator analysis due to the observed statistical heterogeneity among the included studies [47]. Continuous variables including mean age and male and female percentage were used in the meta-regression models. Sub-group analysis was conducted for categorical variables including 1) first responders (EMS personnel, police, and firefighters), 2) continent (America, Asia, Europe, and Africa), 3) sample size (≥500 and <500), 4) study design (cross-sectional and prospective), 5) country status (high-income and middle-income), 6) study quality (moderate and low risk studies), and 7) year of publication (<2020 and ≥2020).

### Ethical approval

This study used secondary data from previously published studies that already sought informed consent from the participants and required no ethical approval.

## RESULTS

### Study characteristics

A total of 1119 studies were retrieved from Psych Info, Web of Science, CINAHL, and PubMed and 28 were included [13-40], published between 2002 and 2022 (**Figure 1**) with 101 080 first responders for medical emergencies including police, EMS personnel, firefighters, and paramedics. Among the 101 080 first responders, 82 840 were males, 8872 were females, and 9368 were identified as other types of gender. A cross-sectional design was used in 26 studies, while two studies used prospective cohort design. Ten studies were done in North America, 11 in Asia, four in Europe, two in South America, and one in Africa. Eight studies had low risk of bias, while 20 studies had moderate risk of bias.

We found six disorders, including insomnia (15 studies), EDS (14 studies), OSA (13 studies), SWD (five studies), restless leg syndrome (two studies), and narcolepsy (two studies). The most common reported sleep disorders were insomnia, EDS, OSA, and SWD and the least reported sleep disorders were restless leg syndrome and narcolepsy. The prevalence of insomnia measured by AIS and ISI ranged from 3.4% to 74.1%. The prevalence of EDS measured by ESS ranged from 17.1% to 44.9%. The prevalence of OSA measured by AHI, BQ, and STOP-BANG questionnaire ranged from 9.9% to 80.8%. The prevalence of SWD measured by SWDSQ and ICSD-2 ranged from 9.1% to 54.0%. The prevalence of restless leg syndrome measured by RLSESTQ ranged from 1.6% to 3.4%. The prevalence of narcolepsy ranged from 0.4% to 4.0%. The associated comorbidities for OSA and insomnia included anxiety, depression, CVD, DM, GERD, and PTSD (**Table 1** and **Table 2**).

**Table 2:** Disease characteristics for sleep disorders among first responders

Author (year)	Type of first responders	Sleep disorder (tool)	Prevalence (N (%))	Prevalence (%)	Associated factors	Associated comorbidities
Angehrn et al. (2020) [13]	Paramedics, police, firefighters	Insomnia (ISI)	418/678/1424	60.0/53.0/55.6	Age, gender, marital status, work experience	NA
Barger et al. (2015) [14]	Firefighters	Insomnia (AIS), OSA (BQ), SWD (ICSD-2), RLS (RLSEST-Q)	416/1969/631/236	6.0/28.4/9.1/3.4	Age, gender, marital status, work experience	Depression, anxiety, DM, CVD
Cramm et al. (2021) [15]	Firefighters	Insomnia (ISI)	256	21.0	Age, gender, marital status, work experience	Depression, anxiety, PTSD
Fernandez et al. (2014) [16]	EMS personnel	EDS (ESS)	394	37.6	Age, gender, marital status, work experience	NA
Garbarino et al. (2002) [17]	Police	EDS (ESS)	243	19.0	Age, gender, marital status, work experience	NA
Garbarino et al. (2019) [18]	Police	EDS (ESS)	42	17.8	Age, gender, marital status, work experience	NA
Glaser et al. (2014) [19]	Firefighters	OSA (AHI, PSG)	514	80.8	Age, gender, marital status, work experience	GERD
Haddock et al. (2013) [20]	Firefighters	EDS (ESS)	125	27.4	Age, gender, marital status, work experience	NA
Hendrickson et al. (2022) [21]	Firefighters, EMS personnel	Insomnia (ISI)	24/40	33.3/34.8	Age, gender, marital status, work experience	NA
Jang et al. (2020) [22]	Firefighters	Insomnia (ISI)	883	9.0	Age, gender, marital status, work experience	Depression, anxiety, PTSD
Khan et al. (2020) [23]	Paramedics	Insomnia (ISI), EDS (ESS), OSA (BQ), SWD (SWDSQ), narcolepsy	56/34/41/67/5	41.5/25.0/30.0/49.0/4.0	Age, gender, marital status, work experience	NA
Khan et al. (2020) [24]	Paramedics	Insomnia (ISI), EDS (ESS), OSA (BQ), SWD (SWDSQ)	28/18/38/45	34.0/22.0/46.0/54.0	Age, gender, marital status, work experience	NA
Khan et al. (2020) [24]	Paramedics	Insomnia (ISI), EDS (ESS), OSA (BQ), SWD (SWDSQ)	53/33/37/53	51.0/32.0/36.0/51.0	Age, gender, marital status, work experience	NA
Kim et al. (2021) [25]	Firefighters	OSA (BQ)	8,236	16.1	Age, gender, marital status, work experience	Depression
Klawe et al. (2005) [26]	Police	OSA (AHI, PSG)	8	8.0	Age, gender, marital status, work experience	NA
Kwak et al. (2020) [27]	Firefighters	Insomnia (ISI)	166	47.2	Age, gender, marital status, work experience	NA
Lim et al. (2020) [28]	Firefighters	Insomnia (ISI)	890	9.1	Age, gender, marital status, work experience	PTSD
Pan et al. (2019) [29]	Police	Insomnia (ISI), EDS (ESS), OSA (BQ)	228/179/103	22.0/17.1/9.9	Age, gender, marital status, work experience	NA
Pazmino Erazo et al. (2020) [30]	Paramedics	Insomnia (ISI)	20	74.1	Age, gender, marital status, work experience	NA
Pinto et al. (2018) [31]	Police	Insomnia (ISI), EDS (ESS), OSA (AHI, PSG)	7/5/6	31.8/22.7/27.3	Age, gender, marital status, work experience	NA
Pirrallo et al. (2012) [32]	EMTs	EDS (ESS)	466	35.7	Age, gender, marital status, work experience	NA
Rajaratnam et al. (2011) [33]	Police	Insomnia (AIS), EDS (ESS)/OSA (BQ)/SWD (SWDSQ)/RLS (RLSEST-Q)/narcolepsy	281/589/1666/269/70/16	6.5/31.6/33.6/14.5/1.6/0.4	Age, gender, marital status, work experience	Depression, anxiety, DM
Savall et al. (2021) [34]	Firefighters	Insomnia (ISI), EDS (ESS), OSA (STOP-BANG)	36/53/3	18.8/27.7/1.3	Age, gender, marital status, work experience	NA
Shi et al., (2021) [35]	Firefighters	EDS (ESS)	112	41.8	Age, gender, marital status, work experience	Depression, anxiety, PTSD
Sofianopoulos et al. (2020) [36]	Paramedics	EDS (ESS), OSA (BQ)	18/13	30.0/21.7	Age, gender, marital status, work experience	NA
Sunderram et al. (2018) [37]	EMS personnel	OSA (BQ)	451	75.0	Age, gender, marital status, work experience	Depression, anxiety, PTSD, DM, GERD, CVD
Tsehay et al. (2021) [38]	Police	Insomnia (ISI)	61	15.8	Age, gender, marital status, work experience	NA
Webber et al. (2011) [39]	EMS personnel	OSA (BQ)	1735	36.5	Age, gender, marital status, work experience	PTSD, GERD
Yadav et al. (2015) [40]	Police	EDS (ESS)	33	38.8	Age, gender, marital status, work experience	NA

AHI – Apnea-Hypopnea index, AIS – Athens Insomnia Scale, BQ – Berlin Questionnaire, CVD – cardiovascular disease, DM – diabetes mellitus, EDS – excessive daytime sleepiness, ESS – Epworth Sleepiness Scale, GERD – gastroesophageal reflux disease, ICSD-2 – International Classification of Sleep Disorders – 2^nd^ Edition, ISI – Insomnia Severity Index, ISS – Insomnia Symptom Scale, N – number, NA – not available, OSA – obstructive sleep apnoea, PTSD – post-traumatic stress disorder, PSG – polysomnography, RLS – restless leg syndrome, RLSESTQ – Restless Leg Syndrome Epidemiology, Symptoms and Treatment Questionnaire, STOP-BANG questionnaire – snoring, tiredness, observed apnoea, blood pressure, body mass index, age, neck size, gender questionnaire, SWD – shift work disorder, SWDSQ – Shift Work Disorder Screening Questionnaire

### Prevalence of shift work disorder among first responders

**Figure 2:**
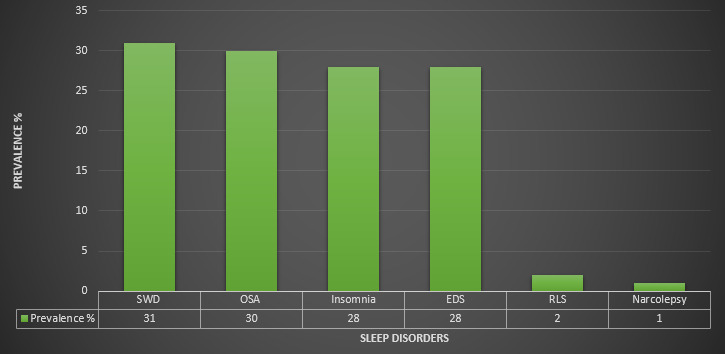
Prevalence of sleep disorders among first responders.

The pooled prevalence of SWD among first responders for medical emergencies was estimated at 31% (95% CI = 15%-53%). We found statistical heterogeneity among the included studies (Q = 361.69, τ^2^ = 1.0802, *I^2^* = 97%; *P* = 0.01) (**Figure 2**, **Table 3**, Figure S1 in the **Online Supplementary Document**).

**Table 3:** Prevalence of sleep disorders and associated comorbidities

				Heterogeneity		
**Sleep disorders**	**n**	**Prevalence (95% CI)**	**N**	***I^2^***	**τ^2^**	***P***
SWD	5	31% (15%-53%)	9117	99%	1.0802	<0.01
OSA	13	30% (18%-46%)	70 687	100%	1.5367	<0.0001
Insomnia	15	28% (19%-39%)	38 518	100%	1.2234	<0.0001
EDS	14	28% (24%-33%)	7905	96%	0.1715	<0.01
RLS	2	2% (1%-4%)	11 231	97%	0.1347	<0.01
Narcolepsy	2	1% (0%-5%)	4434	95%	1.1393	<0.01
**Associated comorbidities**		**OR (95% CI)**	**N**	***I^2^***	**τ^2^**	***P***
OSA						
*Anxiety*	2	2.46 (1.99-3.03)	11 890	0%	0.0000	0.447
*CVD*	2	2.03 (1.43-2.88)	11 890	0%	0.0000	0.896
*DM*	2	1.91 (1.41-2.65)	11 890	0%	0.0000	0.689
*Depression*	4	1.89 (1.01-3.56)	63 640	97%	0.3870	<0.0001
*GERD*	6	1.83 (1.50-2.22)	11 150	48%	0.0220	0.084
*PTSD*	2	1.78 (1.33-2.39)	5557	42%	0.0240	0.188
Insomnia						
*Depression*	2	9.74 (4.67-20.3)	10 955	93%	0.3910	<0.0001
*Anxiety*	2	9.22 (3.81-22.3)	10 955	94%	0.5720	<0.0001
*PTSD*	3	7.13 (6.27-8.10)	20 743	19%	0.0030	0.291

CI – confidence interval, CVD – cardiovascular disease, DM – diabetes mellitus, EDS – excessive daytime sleepiness, GERD – gastroesophageal reflux disease, n – number of studies, N – sample size, OR – odds ratio, OSA – obstructive sleep apnoea, PTSD – post-traumatic stress disorder, RLS – restless leg syndrome, SWD – shift work disorder

### Prevalence of obstructive sleep apnea among first responders

The pooled prevalence of OSA among first responders for medical emergencies was estimated at 30% (95% CI = 18%-46%). We found statistical heterogeneity among the included studies (Q = 4069.89, τ^2^ = 1.5367, *I^2^* = 100%; *P* = 0.0001) (**Figure 2**, **Table 3**, Figure S2 in the **Online Supplementary Document**). The Egger regression co-efficient was 11.1 (t-value = 1.91, *P* = 0.08) and the funnel plot showed no evidence of publication bias (Figure S7 in the **Online Supplementary Document**).

### Prevalence of insomnia among first responders

The pooled prevalence of insomnia among first responders for medical emergencies was estimated at 28% (95% CI = 19%-39%). We observed statistical heterogeneity among the included studies (Q = 599.64, τ^2^ = 1.2234, *I^2^* = 100%; *P* = 0.0001) (**Figure 2**, **Table 3**, Figure S3 in the **Online Supplementary Document**). The Egger regression co-efficient was 13.7 (t-value = 3.66, *P* = 0.002) and the funnel plot showed evidence of publication bias (Figure S8 in the **Online Supplementary Document**).

### Prevalence of excessive daytime sleepiness among first responders

The pooled prevalence of EDS among first responders for medical emergencies was estimated at 28% (95% CI = 24%-33%). We observed statistical heterogeneity among the included studies (Q = 975.41, τ^2^ = 0.1715, *I^2^* = 96%; *P* = 0.01) (**Figure 2**, **Table 3**, Figure S4 in the **Online Supplementary Document**). The Egger regression co-efficient was 1.35 (t-value = 0.52, *P* = 0.614) and the funnel plot showed no evidence of publication bias (Figure S9 in the **Online Supplementary Document**).

### Prevalence of restless leg syndrome among first responders

The pooled prevalence of RLS among first responders for medical emergencies was estimated at 2% (95% CI = 1%-4%). We found statistical heterogeneity among the included studies (Q = 33.90, τ^2^ = 0.1347, *I^2^* = 97% (*P* = 0.01) (**Figure 2**, **Table 3**, Figure S5 in the **Online Supplementary Document**).

### Prevalence of narcolepsy among first responders

The pooled prevalence of narcolepsy among first responders for medical emergencies was estimated at 1% (95% CI = 0%-5%). We found statistical heterogeneity among the included studies (Q = 12.93, τ^2^ = 1.1393, *I^2^* = 95%; *P* = 0.01) (**Figure 2**, **Table 3**, Figure S6 in the **Online Supplementary Document**).

### Associated comorbidities for OSA and insomnia

We found that first responders with OSA were more likely to be at risk of developing anxiety, depression, CVD, DM, GERD, and PTSD compared to first responders without OSA. Regarding mental disorders, first responders with OSA were more likely to be at risk of developing anxiety (OR = 2.46, 95% CI = 1.99-3.03), depression (OR = 1.89, 95% CI = 1.01-3.56), and PTSD (OR = 1.78, 95% CI = 1.33-2.39) compared to first responders without OSA. Regarding physical diseases, first responders with OSA were more likely to be at risk of developing CVD (OR = 2.03, 95% CI = 1.43-2.88), DM (OR = 1.93, 95% CI = 1.41-2.65), and GERD (OR = 1.83, 95% CI = 1.50-2.22) were more likely at risk of OSA compared to first responders without OSA.

We also found that first responders with insomnia were more likely to be at risk of developing depression, anxiety, and PTSD compared to first responders without insomnia. Regarding mental disorders, first responders with insomnia were more likely at risk of developing depression (OR = 9.74, 95% CI = 4.67-20.3), anxiety (OR = 9.22, 95% CI = 3.81-22.3), and PTSD (OR = 7.13, 95% CI = 6.27-8.10) compared to first responders without insomnia (**Table 2**).

### Results of the moderator analysis for OSA, insomnia, and EDS

The results of meta-regression and sub-group analysis for the prevalence of OSA demonstrated that age and continent were significant moderator variables, while first responders, study quality, sample size, study design, country status, and year of publication were not. Regarding age (*P* = 0.049), the study findings show a significant 14% increase in the prevalence of OSA among first responders for medical emergencies. Regarding continent (*P* = 0.049), studies conducted in America found a higher prevalence of OSA (48%) compared to those conducted in Asia (24) and Europe (9%). Regarding year of publication (*P* = 0.160), studies that were conducted before 2020 found a prevalence of 39% compared to a prevalence of 20% found by studies conducted after 2020.

The results of meta-regression and sub-group analysis for the prevalence of insomnia revealed that male percentage, first responders, continent, study quality, sample size, and year of publication were significant moderator variables while age, female percentage, country status, and year of publication were not significant moderator variables. Regarding male percentage (*P* = 0.010), the study findings reveal a 1% decrease in the prevalence of insomnia among first responders for medical emergencies. Regarding continent (P = 0.097), studies conducted in America found a higher prevalence of insomnia (31%) compared to Asia (27%), Europe (19%), and Africa (16%). Regarding, study quality (*P* = 0.0001), moderate risk studies found a prevalence of 34% for insomnia compared to 11% found in low-risk studies. Regarding sample size (*P* = 0.089), studies with sample sizes <500 found a prevalence of 37% for insomnia, while studies with sample sizes ≥500 found a prevalence of 21%. Studies that used a prospective cohort design found a prevalence of 6% for insomnia compared to a prevalence of 30% found by studies using a cross-sectional design. Regarding year of publication (P = 0.013), studies conducted before 2020 found a prevalence of 36% for insomnia compared to a prevalence of 14% found by studies conducted after 2020.

The results of meta-regression and sub-group analysis for the prevalence of EDS revealed that male percentage and continent were significant moderator variables while age, first responders, female percentage, study quality, sample size, country status, and assessment tool were not. Regarding male percentage (*P* = 0.049), the study findings reveal a 1% decrease in the prevalence of EDS among first responders for medical emergencies. Regarding continent (*P* = 0.0001), studies conducted in America found a higher prevalence of EDS (36%) compared to Asia (26) and Europe (21%). Regarding year of (*P* = 0.445), studies that were conducted before 2020 found a prevalence of 28% for EDS compared to a prevalence of 31% in studies conducted after 2020 (Table S4 in the **Online Supplementary Document**).

## DISCUSSION

### Prevalence and associated comorbidities of sleep disorders among first responders

To our knowledge, this is the first meta-analysis to provide comprehensive evidence on the prevalence of sleep disorders (including SWD, OSA, insomnia, EDS, RLS, and narcolepsy) and their associated comorbidities among first responders for medical emergencies. The current study findings show that a considerable number of first responders suffer from SWD (31%), OSA (30%), insomnia (28%), and EDS (28%) compared to RLS (2%) and narcolepsy (1%). Moreover, first responders with OSA were more likely to develop anxiety, depression, CVD, DM, GERD, and PTSD while first responders with insomnia were more likely to develop depression, anxiety, and PTSD. Furthermore, the study findings show that age, gender, first responders, continent, study quality, and study design for OSA, insomnia, and EDS could partially explain the variation among the included studies.

Our findings show similar prevalence rates of sleep disturbances among first responders to that among other healthcare workers, including physicians, nurses, and allied health professionals [48,49] and higher prevalence rates compared to those of the general population [50]. Qiu et al. [48] revealed that the pooled prevalence of sleep disturbances was estimated at 39% among Chinese health professionals while Xia et al. [49] showed that the pooled prevalence of sleep disturbances among Chinese doctors and nurses and doctors was 35% and 43%, respectively. Sleep and the timing of sleep are regulated by the circadian rhythms and sleep homeostasis process through the regulation of cortisol and melatonin levels, with cortisol levels in shift workers being higher during sleep and lower when awake compared to levels in regular daytime workers [51,52]. Shift workers’ melatonin levels are lower during sleep compared to those of regular daytime workers, and the hormonal imbalance between cortisol and melatonin levels leads to the development of sleep problems in shift workers, including first responders. As such, first responders (owing to the nature of their jobs with 24-hour shifts) are likely to have high prevalence rates of sleep problems including SWD, OSA, insomnia, and EDS as observed in the current meta-analysis. Thus, early assessment of sleep disorders among first responders is crucial to provide appropriate interventions that can help to prevent development of physical and mental problems including anxiety, CVD, DM, depression, GERD, and PTSD. Additionally, we suggest efficient allocation of shifts among first responders to help maintain good and quality sleep leading to better regulation of the circadian rhythm and sleep homeostasis process preventing development of sleep disorders including SWD, OSA, insomnia, EDS, RLS, and narcolepsy.

The current meta-analysis found that OSA was associated with risk of anxiety, CVD, DM, depression, GERD, and PTSD, while insomnia was associated with risk of depression, anxiety, and PTSD. These findings agree with those of previous studies that found that sleep disturbances 8including insomnia and OSA) were associated with high risk of diabetes mellitus, obesity CVD, hypertension, stroke, depression, and anxiety [53,54]. Shift work and irregular work schedules have been associated with the disturbance of the circadian rhythm and sleep function [7,55]. Moreover, the disturbance of circadian rhythm has been associated with high risk of mental health problems (including anxiety, depression, and PTSD, CVD, DM) and gastrointestinal problems (including GERD) [55]. As such, the likelihood of developing physical and mental health problems (including anxiety, CVD, DM, depression, GERD, and PTSD) among first responders with sleep disorders is high. Thus, increased efforts among first responders for medical emergencies should be taken to examine associated comorbidities of sleep disorders including mental health problems (including anxiety, depression, and PTSD, CVD, DM) and gastrointestinal problems (including GERD). Moreover, management of these comorbidities and ensuring and maintaining good and quality sleep among first responders is crucial for improving their quality of life and productivity. Furthermore, some of the included studies did not use adjusted models of the associated comorbidities of sleep disorders, which may likely underestimate or overestimate these associations; no causal links could be established, so future high quality observational studies with detailed demographic and occupational characteristics are needed to clarify these associations.

The current findings revealed that significant moderator variables for OSA, insomnia, and EDS were age, gender, first responders, continent, study quality, and study design. We also found an increase in the prevalence of OSA with increased age. Evidence suggests that the prevalence of OSA increases with age until the age of 65 years [56]. Therefore, a high prevalence of OSA among these first responders can be expected, as responders in most included studies had an average age of between 30 and 40 years. Gender differences were observed, as male participants demonstrated a decreasing prevalence of insomnia and EDS. Previous study findings have shown that women have a higher risk for insomnia and EDS compared to men [57,58]. Moreover, greater gender disparity has been observed between men and women, as more research on sleep has been conducted in men and the current meta-analysis had 82 840 males compared to 8,872 females. More research in women is needed to clarify this association. Regarding continents, studies conducted in America demonstrated to have higher prevalence of OSA, insomnia, and EDS, followed by Asia and the Europe. The possible explanation of these observed rates could be due to regional culture differences on sleep pattern, socio-economic status, and limited number of studies included in the current meta-analysis. Interestingly, we did not find any difference in the prevalence rates of OSA, insomnia, and EDS when we compared the country status using high-income and middle-income countries.

This meta-analysis has several strengths. It is the first to provide comprehensive evidence on the prevalence of sleep disorders (including SWD, OSA, insomnia, EDS, RLS, and narcolepsy) among first responders for medical emergencies. The included studies were not limited by year of publication or language, and we included studies that used validated assessment tools. Standard reporting for research transparency and study integrity followed the MOOSE guidelines and study protocol registration in PROSPERO. However, some limitations should be considered. First responders (including EMS personnel, firefighters, and police) play different roles when providing pre-hospital medical services, which may likely impact their work schedules and disturbance of their normal sleep habits. Second, most of the included studies used a cross-sectional design, so no causal relationship could be determined as regarding associated comorbidities for OSA and insomnia. Third, statistical heterogeneity in the prevalence estimates of SWD, OSA, insomnia, EDS, RLS, and narcolepsy was observed, and moderator analysis was conducted to explore and explain the sources of heterogeneity. Additionally, the observed differences in the prevalence rates could be attributed to the use of different objective and subjective assessment tools when examining sleep disorders. As such, establishing standard protocols using validated assessment tools for examining sleep disorders among first responders for medical emergencies is necessary to ensure accurate and early detection of the sleep disorders.

## CONCLUSIONS

The current meta-analysis provides comprehensive evidence that the prevalence of sleep disorders is 31% for SWD, 30% for OSA, 28% for insomnia and EDS, 2% for RLS, and 1% for narcolepsy. Anxiety, depression, CVD, DM, GERD, and PTSD were associated comorbidities for OSA and insomnia. Furthermore, significant moderator variables for OSA, insomnia, and EDS were age, gender, first responders, continent, study quality, study design, and assessment tool. Early assessment and management of sleep disorders among first responders for medical emergencies is necessary to promote mechanisms to improve good and quality sleep to help prevent anxiety, depression, CVD, DM, GERD, and PTSD.

## Additional material:


Online Supplementary Document
