# Supplementary material for: Prevalence of sleep disorders among first responders for medical emergencies: A meta-analysis
Source: J Glob Health. 2022 Oct 20;12:04092. doi: 10.7189/jogh.12.04092 (PMC9585923; doi:10.7189/jogh.12.04092)

Prevalence of Sleep Disorders among First Responders for Medical Emergencies: A Meta-Analysis

Garry Huang, Tso-Ying Lee, Kondwani Joseph Banda, Li-Chung Pien, Hsiu-Ju Jen, Ruey Chen, Doresses Liu, Shu-Tai Shen Hsiao, Kuei-Ru Chou

Online Supplementary Document

Table S1: Search Strategy……………………………………………………………………..…2

Table S2: PICOs Criteria…………………………………………………………..……..……5

Table S3: Risk of Bias Assessment………………………………………………..……..……6

Table S4: Moderator Analysis…………..…………………………………………...…….8

Figure S1: Prevalence of Shift Work Disorder……..…………..9

Figure S2: Prevalence of Obstructive Sleep Apnea……...…………..……………..…….……9

Figure S3: Prevalence of Insomnia……...…………..10

Figure S4: Prevalence of Excessive Daytime Sleepiness……...............................…………..10

Figure S5: Prevalence of Restless Leg Syndrome……..…………..11

Figure S6: Prevalence of Narcolepsy……..…………..11

Figure S7: Funnel Plot for Obstructive Sleep Apnea ……...................................…………..12

Figure S8: Funnel Plot for Insomnia ……...………..12

Figure S9: Funnel Plot for Excessive Daytime Sleepiness ……............................…………..13

Table S1: Search Strategy

Database	Keywords and Mesh-Terms	Number of studies
CINAHL	TX (prevalence OR incidence OR epidemiology OR rate OR rates OR number OR proportion OR probability OR event) AND TX (Insomnia OR Narcolepsy OR REM sleep behavior disorder OR restless leg syndrome OR Parasomnias OR Sleep apnea OR Non-24 hour sleep wake disorder OR excessive sleepiness OR shift wake disorder OR periodic limb movement disorder) AND TX (Emergency Medical Services Personnel OR EMS personnel OR ambulance personnel OR fire fighters OR law enforcement OR police OR first responders OR paramedics OR emergency medical technicians OR EMTs)	183
PubMed	("epidemiology"[MeSH Subheading] OR "epidemiology"[All Fields] OR "prevalence"[All Fields] OR "prevalence"[MeSH Terms] OR "prevalance"[All Fields] OR "prevalences"[All Fields] OR "prevalence s"[All Fields] OR "prevalent"[All Fields] OR "prevalently"[All Fields] OR "prevalents"[All Fields] OR ("epidemiology"[MeSH Subheading] OR "epidemiology"[All Fields] OR "incidence"[All Fields] OR "incidence"[MeSH Terms] OR "incidences"[All Fields] OR "incident"[All Fields] OR "incidents"[All Fields]) OR ("epidemiologies"[All Fields] OR "epidemiology"[MeSH Subheading] OR "epidemiology"[All Fields] OR "epidemiology"[MeSH Terms] OR "epidemiology s"[All Fields]) OR ("j rehabil assist technol eng"[Journal] OR "rate"[All Fields]) OR "rates"[All Fields] OR ("number"[All Fields] OR "numbers"[All Fields]) OR ("proportion"[All Fields] OR "proportions"[All Fields]) OR ("probability"[MeSH Terms] OR "probability"[All Fields] OR "probabilities"[All Fields]) OR ("event"[All Fields] OR "event s"[All Fields] OR "events"[All Fields])) AND ("insomnia s"[All Fields] OR "sleep initiation and maintenance disorders"[MeSH Terms] OR ("sleep"[All Fields] AND "initiation"[All Fields] AND "maintenance"[All Fields] AND "disorders"[All Fields]) OR "sleep initiation and maintenance disorders"[All Fields] OR "insomnia"[All Fields] OR "insomnias"[All Fields] OR ("narcolepsy"[MeSH Terms] OR "narcolepsy"[All Fields] OR "narcolepsies"[All Fields]) OR ("rem sleep behaviour disorder"[All Fields] OR "rem sleep behavior disorder"[MeSH Terms] OR ("rem"[All Fields] AND "sleep"[All Fields] AND "behavior"[All Fields] AND "disorder"[All Fields]) OR "rem sleep behavior disorder"[All Fields]) OR ("restless legs syndrome"[MeSH Terms] OR ("restless"[All Fields] AND "legs"[All Fields] AND	303

	"syndrome"[All Fields]) OR "restless legs syndrome"[All Fields] OR ("restless"[All Fields] AND "leg"[All Fields] AND "syndrome"[All Fields]) OR "restless leg syndrome"[All Fields]) OR ("parasomnias"[MeSH Terms] OR "parasomnias"[All Fields] OR "parasomnia"[All Fields]) OR ("sleep apnoea"[All Fields] OR "sleep apnea syndromes"[MeSH Terms] OR ("sleep"[All Fields] AND "apnea"[All Fields] AND "syndromes"[All Fields]) OR "sleep apnea syndromes"[All Fields] OR ("sleep"[All Fields] AND "apnea"[All Fields]) OR "sleep apnea"[All Fields]) OR ("sleep disorders, circadian rhythm"[MeSH Terms] OR ("sleep"[All Fields] AND "disorders"[All Fields] AND "circadian"[All Fields] AND "rhythm"[All Fields]) OR "circadian rhythm sleep disorders"[All Fields] OR "non 24 hour sleep wake disorder"[All Fields]) OR ("disorders of excessive somnolence"[MeSH Terms] OR ("disorders"[All Fields] AND "excessive"[All Fields] AND "somnolence"[All Fields]) OR "disorders of excessive somnolence"[All Fields] OR ("excessive"[All Fields] AND "sleepiness"[All Fields]) OR "excessive sleepiness"[All Fields]) OR (("shift"[All Fields] OR "shifted"[All Fields] OR "shifting"[All Fields] OR "shiftings"[All Fields] OR "shifts"[All Fields]) AND "wake"[All Fields] AND ("disease"[MeSH Terms] OR "disease"[All Fields] OR "disorder"[All Fields] OR "disorders"[All Fields] OR "disorder s"[All Fields] OR "disordes"[All Fields])) OR ("nocturnal myoclonus syndrome"[MeSH Terms] OR ("nocturnal"[All Fields] AND "myoclonus"[All Fields] AND "syndrome"[All Fields]) OR "nocturnal myoclonus syndrome"[All Fields] OR ("periodic"[All Fields] AND "limb"[All Fields] AND "movement"[All Fields] AND "disorder"[All Fields]) OR "periodic limb movement disorder"[All Fields])) AND ((("emergency medical services"[MeSH Terms] OR ("emergency"[All Fields] AND "medical"[All Fields] AND "services"[All Fields]) OR "emergency medical services"[All Fields]) AND ("occupational groups"[MeSH Terms] OR ("occupational"[All Fields] AND "groups"[All Fields]) OR "occupational groups"[All Fields] OR "personnel"[All Fields] OR "personnel s"[All Fields] OR "personnels"[All Fields])) OR (("emerg med serv"[Journal] OR "ems mag"[Journal] OR "ems"[All Fields]) AND ("occupational groups"[MeSH Terms] OR ("occupational"[All Fields] AND "groups"[All Fields]) OR "occupational groups"[All Fields] OR "personnel"[All Fields] OR "personnel s"[All Fields] OR "personnels"[All Fields])) OR (("ambulance s"[All Fields] OR "ambulances"[MeSH Terms] OR "ambulances"[All Fields] OR	

	"ambulance"[All Fields]) AND ("occupational groups"[MeSH Terms] OR ("occupational"[All Fields] AND "groups"[All Fields]) OR "occupational groups"[All Fields] OR "personnel"[All Fields] OR "personnel s"[All Fields] OR "personnels"[All Fields])) OR ("firefighters"[MeSH Terms] OR "firefighters"[All Fields] OR ("fire"[All Fields] AND "fighters"[All Fields]) OR "fire fighters"[All Fields]) OR ("law enforcement"[MeSH Terms] OR ("law"[All Fields] AND "enforcement"[All Fields]) OR "law enforcement"[All Fields]) OR ("police"[MeSH Terms] OR "police"[All Fields] OR "polices"[All Fields] OR "police s"[All Fields] OR "policed"[All Fields] OR "policing"[All Fields]) OR ("emergency responders"[MeSH Terms] OR ("emergency"[All Fields] AND "responders"[All Fields]) OR "emergency responders"[All Fields] OR ("first"[All Fields] AND "responders"[All Fields]) OR "first responders"[All Fields]) OR ("allied health personnel"[MeSH Terms] OR ("allied"[All Fields] AND "health"[All Fields] AND "personnel"[All Fields]) OR "allied health personnel"[All Fields] OR "paramedics"[All Fields] OR "emergency medical technicians"[MeSH Terms] OR ("emergency"[All Fields] AND "medical"[All Fields] AND "technicians"[All Fields]) OR "emergency medical technicians"[All Fields] OR "paramedic"[All Fields] OR "paramedic s"[All Fields] OR "paramedical"[All Fields] OR "paramedicals"[All Fields]) OR ("emergency medical technicians"[MeSH Terms] OR ("emergency"[All Fields] AND "medical"[All Fields] AND "technicians"[All Fields]) OR "emergency medical technicians"[All Fields]) OR "EMTs"[All Fields])	
Psych Info	TX (prevalence OR incidence OR epidemiology OR rate OR rates OR number OR proportion OR probability OR event) AND TX (Insomnia OR Narcolepsy OR REM sleep behavior disorder OR restless leg syndrome OR Parasomnias OR Sleep apnea OR Non-24 hour sleep wake disorder OR excessive sleepiness OR shift wake disorder OR periodic limb movement disorder) AND TX (Emergency Medical Services Personnel OR EMS personnel OR ambulance personnel OR fire fighters OR law enforcement OR police OR first responders OR paramedics OR emergency medical technicians OR EMTs)	67
Web of Science	prevalence OR incidence OR epidemiology OR rate OR rates OR number OR proportion OR probability OR event **(All Fields)** **and** Insomnia OR Narcolepsy OR REM sleep behavior disorder OR restless leg syndrome OR Parasomnias OR Sleep apnea OR Non-24 hour sleep wake disorder OR excessive sleepiness OR shift wake disorder OR periodic limb movement disorder **(All Fields)** **and** Emergency Medical Services	566

	Personnel OR EMS personnel OR ambulance personnel OR fire fighters OR law enforcement OR police OR first responders OR paramedics OR emergency medical technicians OR eats **(All Fields)**<br>Timespan: All years. Indexes: SCI-EXPANDED, SSCI.	

Table S2: PICOs Criteria

Population	First responders including emergency medical service (EMS) personnel, ambulance personnel, paramedics, police, emergency medical technicians (EMTs), and fire fighters
Exposure	Sleep disorders (insomnia, narcolepsy, REM sleep behavior disorder, restless leg syndrome, parasomnias, sleep apnea, non-24-hour sleep wake disorder, excessive sleepiness, shift wake disorder, periodic limb movement disorder)
Comparison	No sleep disorders
Outcome	epidemiology, incidence, prevalence
Study design	observational studies including cross-sectional and prospective studies

Table S3: Risk of Bias Assessment

Study	External validity				Internal validity						Total
	**Representativeness**	**Sampling frame**	**Random selection**	**Non-response**	**Data collection**	**Case definition**	**Instrument**	**Same mode**	**Prevalence period**	**Numerator / denominator**	
Angehrn, 2020	1	1	0	0	1	1	1	1	1	1	8 – M
Barger, 2015	1	1	0	1	1	1	1	1	1	1	9 – L
Cramm, 2021	1	1	0	1	0	1	1	1	1	1	8 – M
Fernandez, 2020	1	1	1	1	1	1	1	1	1	1	10 – L
Garbarino, 2002	1	1	0	1	0	1	1	1	1	1	7 – M
Garbarino, 2019	0	0	0	1	1	1	1	1	1	1	7 – M
Glaser, 2014	1	0	0	1	1	1	1	1	1	1	8 – M
Haddock, 2013	0	0	1	1	1	1	1	1	1	1	8 – M
Hendrickson, 2022	0	0	0	1	1	1	1	1	1	1	7 – M
Jang, 2019	1	1	0	1	1	1	1	1	1	1	9 – L
Khan, 2019	0	0	0	1	1	1	1	1	1	1	7 – M
Khan, 2020	0	0	0	1	1	1	1	1	1	1	7 – M
Kim, 2021	1	1	0	1	1	1	1	1	1	1	9 – L
Klawe, 2005	0	0	0	1	1	1	1	1	1	1	7 – M
Kwak, 2020	1	0	0	1	1	1	1	1	1	1	8 – M
Lim, 2020	1	1	0	1	1	1	1	1	1	1	9 – L
Pan, 2019	1	1	0	1	1	1	1	1	1	1	9 – L
Pazmino-Erazo, 2020	0	0	0	1	1	1	1	1	1	1	7 – M
Pinto, 2018	0	0	0	1	1	1	1	1	1	1	7 – M
Pirrallo, 2012	1	0	0	0	1	1	1	1	1	1	7 – M
Rajaratnam, 2011	1	1	0	1	1	1	1	1	1	1	9 – L
Savall, 2021	0	0	0	1	1	1	1	1	1	1	7 – M
Shi, 2020	1	0	0	0	1	1	1	1	1	1	7 – M

Study	External validity				Internal validity						Total
	Representativeness	Sampling frame	Random selection	Non-response	Data collection	Case definition	Instrument	Same mode	Prevalence period	Numerator / denominator	
Sofianopolous, 2011	0	0	0	1	1	1	1	1	1	1	7 – M
Sunderram, 2018	1	1	0	1	1	1	1	1	1	1	9 – L
Tsehay, 2021	1	0	0	1	1	1	1	1	1	1	8 – M
Webber, 2011	1	0	0	1	0	1	1	1	1	1	7 – M
Yadav, 2015	0	0	0	1	1	1	1	1	1	1	7 – M

Score of 1 for low risk and 0 for high risk. Poor quality: score of ≤ 6 (H), moderate quality: score of 7 or 8 (M), high quality: score of 9 or 10 (L)

Table S4: Moderator Analysis for OSA, insomnia, and EDS

OSA				Insomnia				EDS			
Variables	P-value	n	Prevalence (95%CI)	Variables	P-value	n	Prevalence (95%CI)	Variables	P-value	n	Prevalence (95%CI)
**age**	**0.049**	14	0.14 (0.00–0.28)	**age**	0.802	19	-0.04 (-0.38–0.29)	**age**	0.966	15	-0.00 (-0.06–0.05)
**Male %**	0.744	14	0.00 (-0.02–0.03)	**Male %**	**0.010**	19	-0.01 (-0.03–(-0.00)	**Male %**	**0.049**	15	-0.01 (-0.02–(-0.00)
**Female %**	0.299	14	-0.02 (-0.07–0.02)	**Female %**	0.673	19	-0.01 (-0.03–0.04)	**Female %**	0.073	15	-0.00 (-0.06–0.05)
**First responders**	0.223	14		**First responders**	**0.002**	19		**First responders**	0.121	15	
EMS personnel		6	0.41 (0.27–0.56)	EMS personnel		6	0.48 (0.38–0.59)	EMS personnel		6	0.32 (0.26–0.40)
Police		4	0.24 (0.13–0.39)	Police		5	0.22 (0.10–0.33)	Police		6	0.23 (0.18–0.30)
Firefighters		4	0.21 (0.04–0.66)	Firefighters		8	0.19 (0.11–0.41)	Firefighters		3	0.32 (0.25–0.40)
**Continent**	**0.049**	14		**Continent**	**0.097**	19		**Continent**	**<0.0001**	15	
America		6	0.48 (0.29–0.67)	America		10	0.31 (0.17–0.50)	America		6	0.36 (0.30–0.41)
Asia		6	0.24 (0.15–0.36)	Asia		7	0.27 (0.15–0.42)	Asia		6	0.26 (0.20–0.33)
Europe		2	0.09 (0.01–0.56)	Europe		1	0.19 (0.14–0.25)	Europe		3	0.21 (0.17–0.25)
Africa		0	NA	Africa		1	0.16 (0.13–0.20)	Africa		0	NA
**Study quality**	0.937	14		**Study quality**	**<0.0001**	19		**Study quality**	0.917	15	
Moderate risk		9	0.30 (0.15–0.52)	Moderate risk		15	0.34 (0.24–0.47)	Moderate risk		12	0.29 (0.24–0.34)
Low risk		5	0.29 (0.14–0.53)	Low risk		4	0.11 (0.09–0.16)	Low risk		3	0.28 (0.19–0.39)
**Sample size**	0.945	14		**Sample size**	**0.089**	19		**Sample size**	0.534	15	
>500		5	0.31 (0.13–0.57)	>500		10	0.21 (0.11–0.36)	>500		6	0.27 (0.19–0.36)
<500		9	0.30 (0.16–0.50)	<500		9	0.37 (0.26–0.48)	<500		9	0.30 (0.26–0.35)
**Study design**	0.514	14		**Study design**	**<0.0001**	19		**Study design**	0.219	15	
Prospective		2	0.35 (0.33–0.37)	Prospective		1	0.06 (0.05–0.07)	Prospective		1	0.32 (0.23–0.33)
Cross-sectional		12	0.29 (0.16–0.48)	Cross-sectional		18	0.30 (0.20–0.41)	Cross-sectional		14	0.28 (0.19–0.34)
**Country status**	0.559	14		**Country status**	0.535	19		**Country status**	0.386	15	
High-income		8	0.33 (0.15–0.59)	High-income		12	0.25 (0.15–0.39)	High-income		10	0.30 (0.24–0.36)
Middle-income		6	0.26 (0.16–0.39)	Middle-income		7	0.32 (0.18–0.50)	Middle-income		5	0.25 (0.19–0.34)
**Publication year**	0.160	14		**Publication year**	**0.013**	19		**Publication year**	0.445	15	
< 2020		8	0.39 (0.23–0.58)	< 2020		13	0.36 (0.26–0.48)	< 2020		10	0.28 (0.22–0.34)
≥ 2020		6	0.20 (0.08–0.41)	≥ 2020		6	0.14 (0.06–0.27)	≥ 2020		5	0.31 (0.25–0.38)

CI: confidence interval, GAD: Generalized Anxiety Disorder, n: number of studies, p-value: probability value, PHQ: Patient Health Questionnaire

Figure S1: Prevalence of Shift Work Disorder

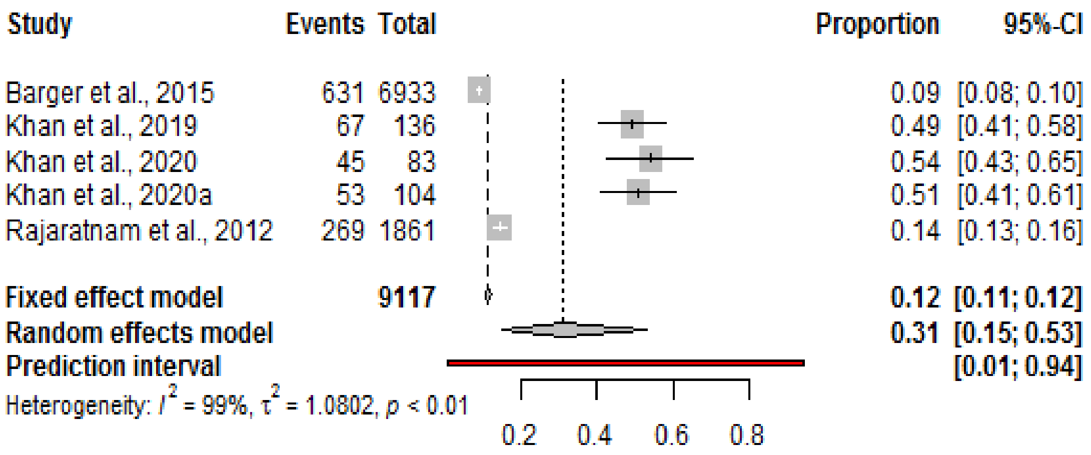

Figure S2: Prevalence of Obstructive Sleep Apnea

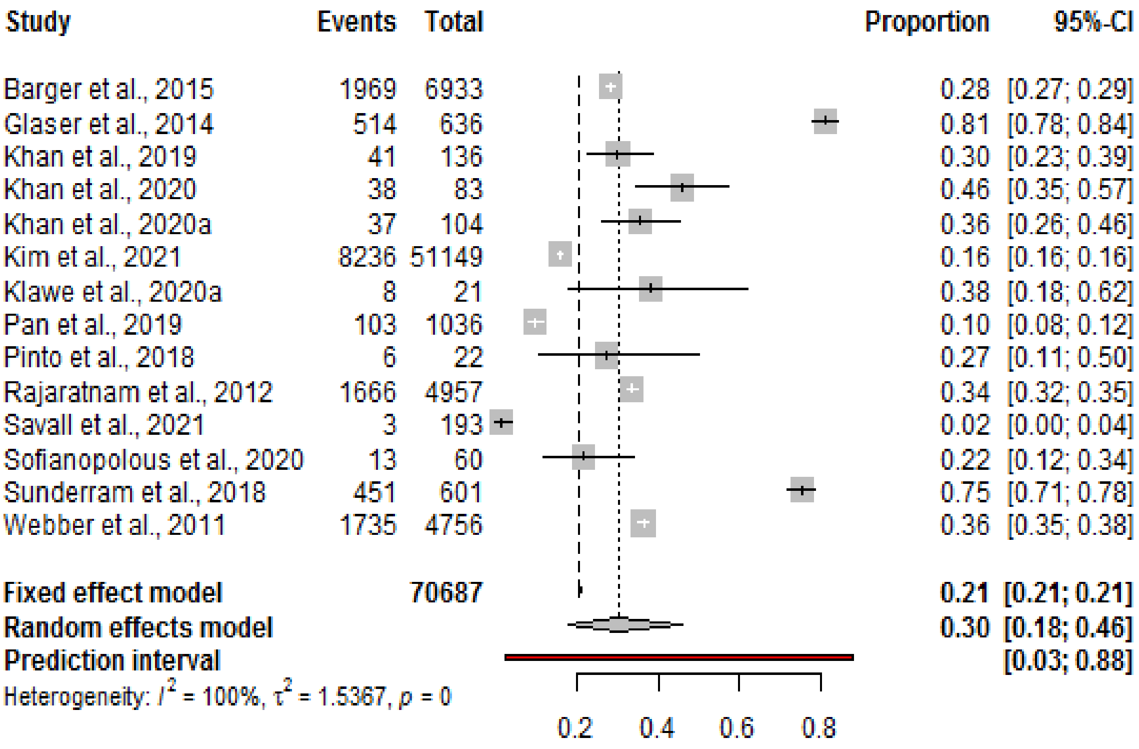

Figure S3: Prevalence of Insomnia

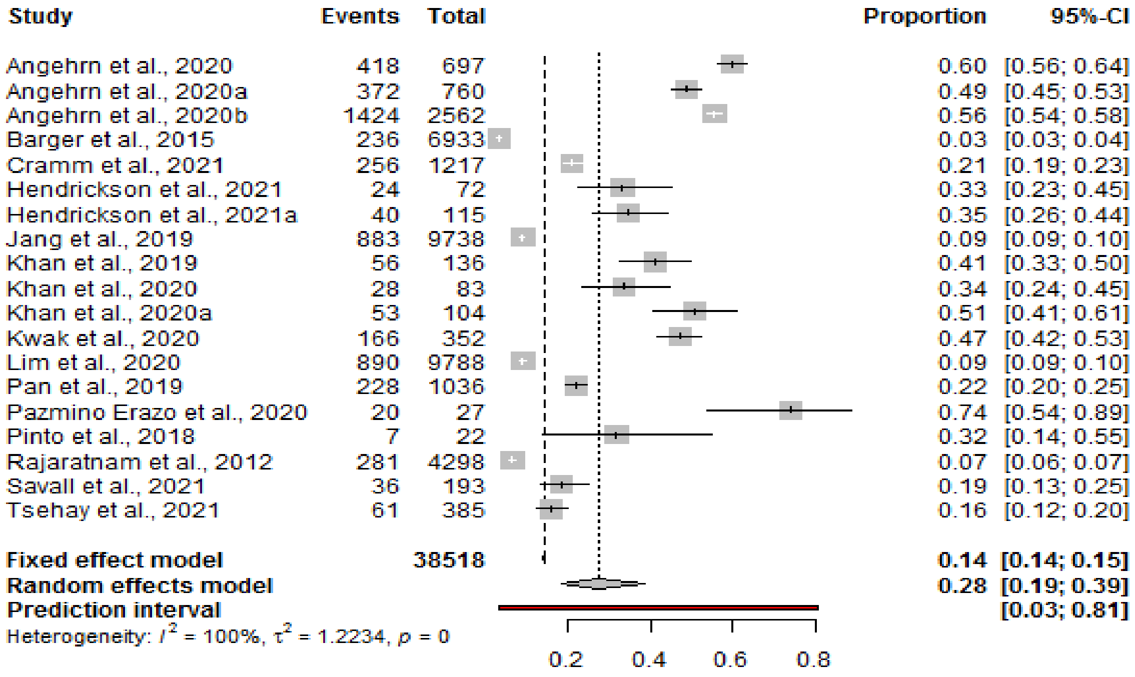

Figure S4: Prevalence of Excessive Daytime Sleepiness

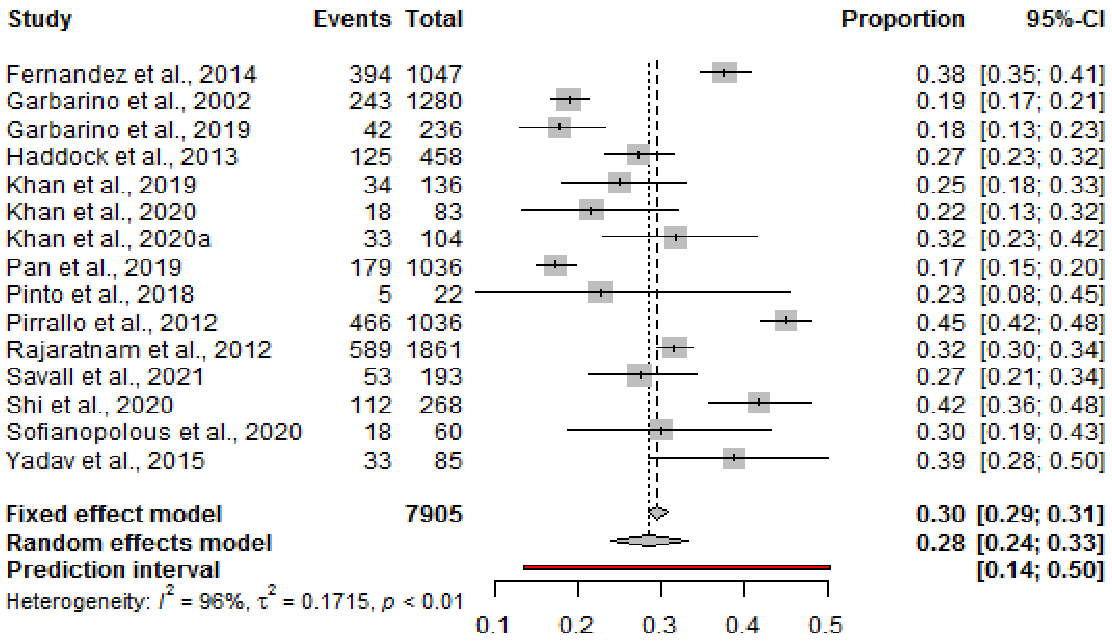

Figure S5: Prevalence of Restless Leg Syndrome

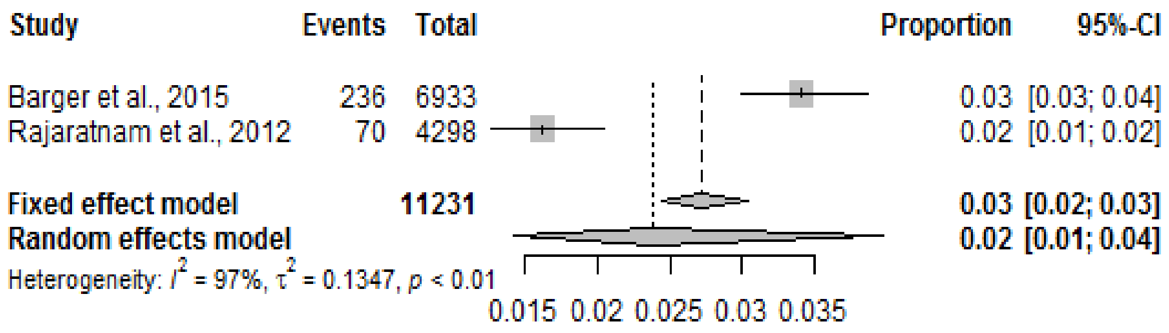

Figure S6: Prevalence of Narcolepsy

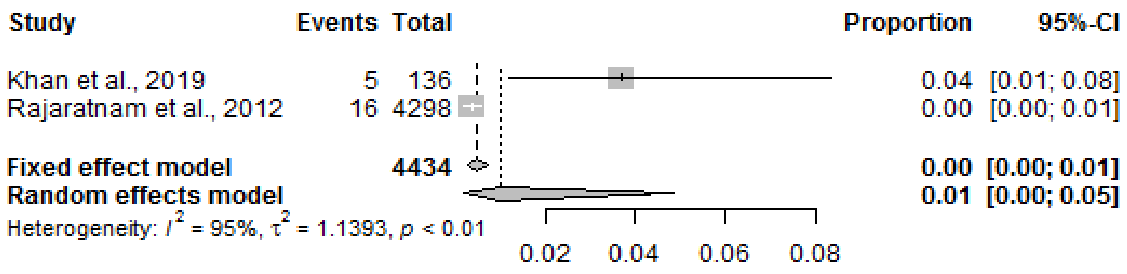

Figure S7: Funnel Plot for Obstructive Sleep Apnea

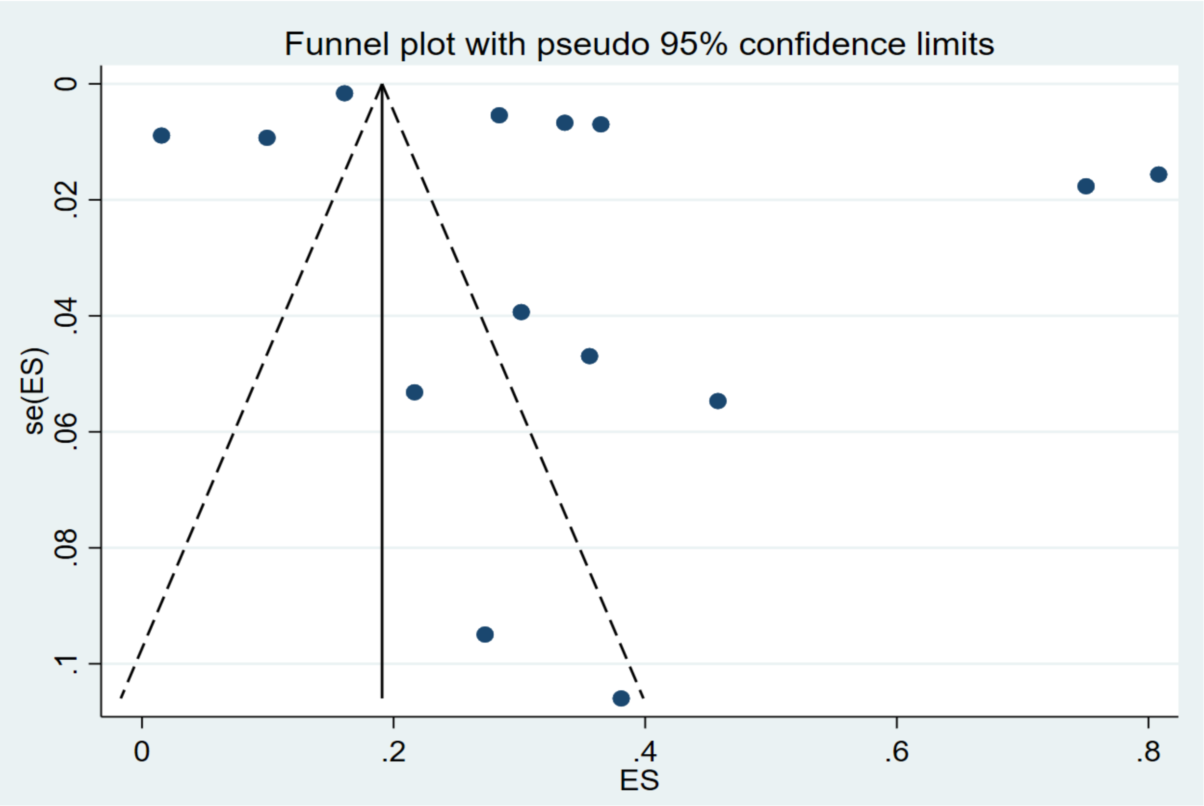

Figure S8: Funnel Plot for Insomnia

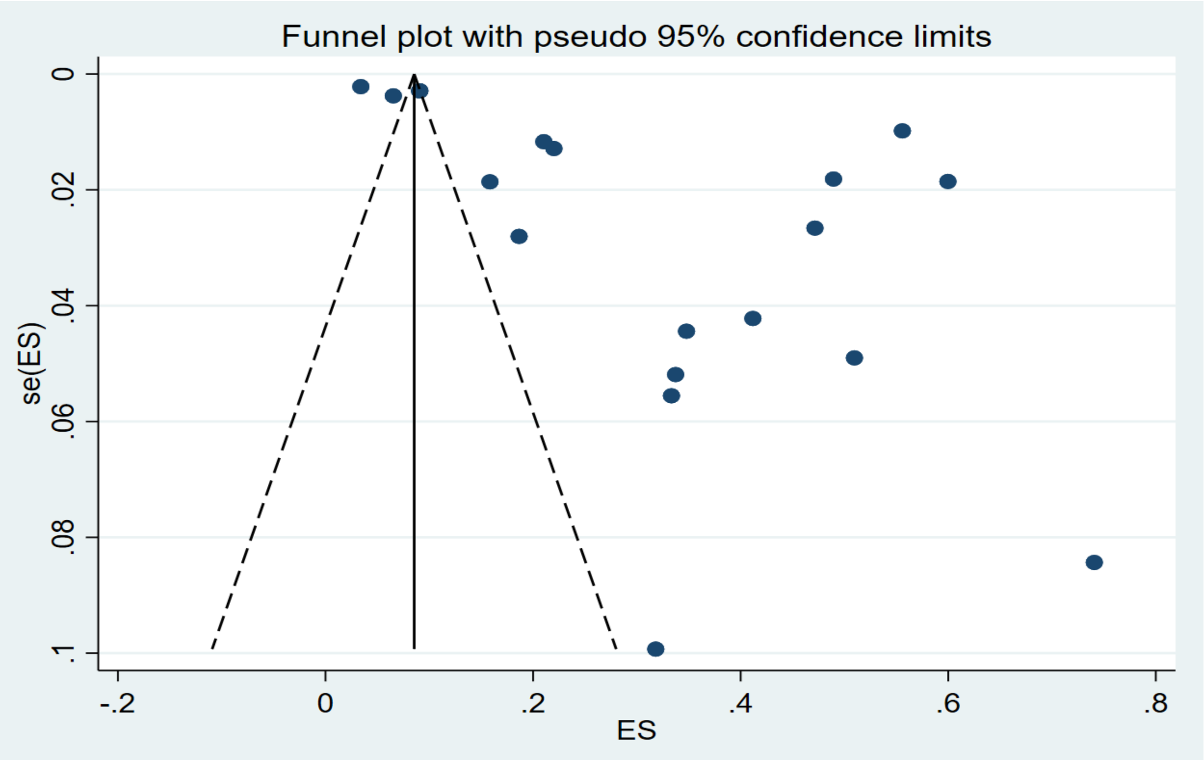

Figure S9: Funnel Plot for Excessive Daytime Sleepiness

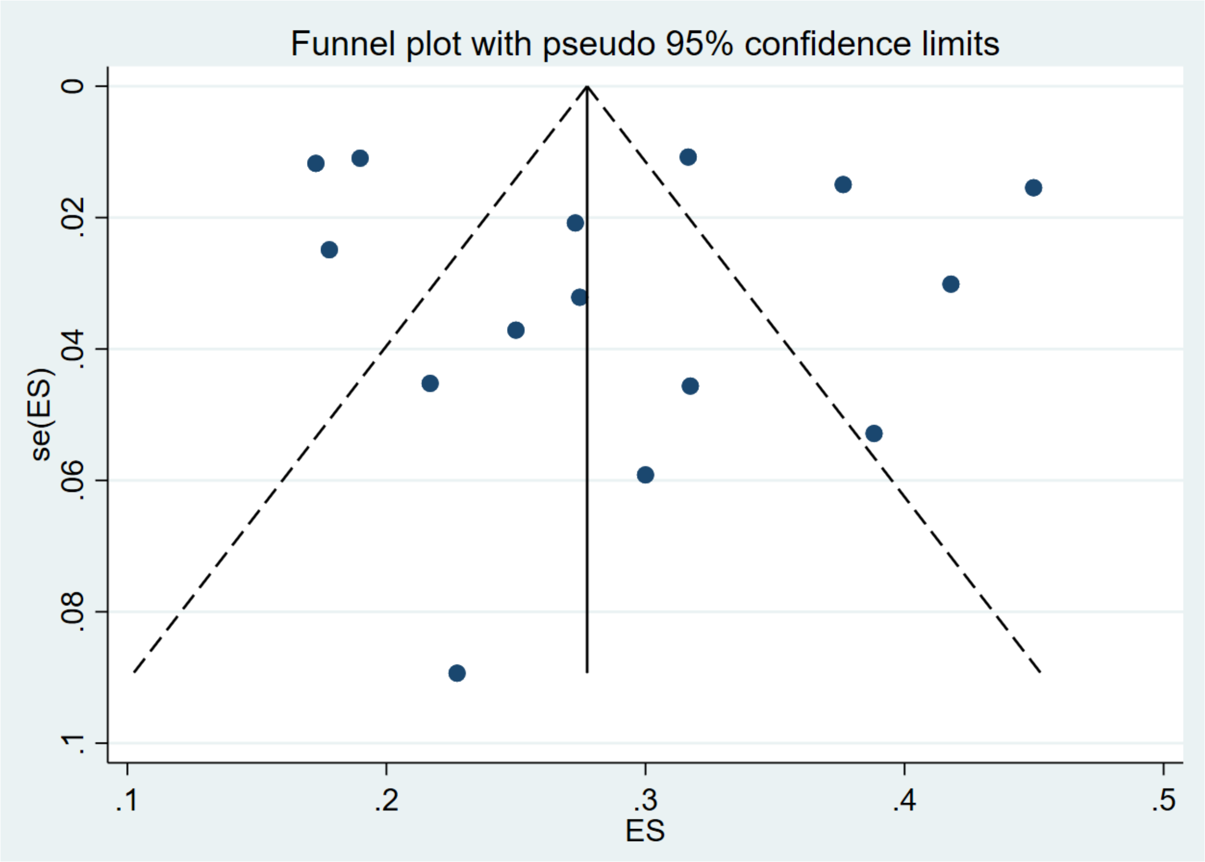

Supplement: Online Supplementary Document [file jogh-12-04092-s001.pdf]
